# Percutaneous Coronary Intervention Outcomes in Patients with Liver Cirrhosis: A Systematic Review and Meta-Analysis

**DOI:** 10.3390/jcdd10030092

**Published:** 2023-02-21

**Authors:** Harshwardhan Khandait, Vikash Jaiswal, Muhammad Hanif, Abhigan Babu Shrestha, Alisson Iturburu, Maitri Shah, Angela Ishak, Vamsi Garimella, Song Peng Ang, Midhun Mathew

**Affiliations:** 1 Trinitas Regional Medical Center/RWJ Barnabas Health, Elizabeth, NJ 07202, USA; 2 Department of Research, JCCR Cardiology Research, Varanasi 221005, India; 3 Department of Internal Medicine, SUNY Upstate Medical University, Syracuse, NY 13210, USA; 4 Department of Medicine, M Abdur Rahim Medical College, Dinajpur 5200, Bangladesh; 5 Department of Medicine, Universidad de Guayaquil, Guayas 090514, Ecuador; 6 Department of Research and Academic Affairs, Larkin Community Hospital, South Miami, FL 33143, USA; 7 Department of Internal Medicine, University of Miami (Holy Cross), Miami, FL 33136, USA; 8 Division of Internal Medicine, Rutgers Health/Community Medical Center, Toms River, NJ 08755, USA

**Keywords:** cirrhosis, coronary artery disease, revascularization

## Abstract

There is a paucity of data and minimal literature on outcomes of percutaneous coronary intervention (PCI) among liver cirrhosis patients. Therefore, we conducted a systematic review and meta-analysis to evaluate the clinical outcomes among liver cirrhosis patients post-PCI. We conducted a comprehensive literature search in the PubMed, Embase, Cochrane, and Scopus databases for relevant studies. Effect sizes were pooled using the DerSimonian and Laird random-effects model as an odds ratio (OR) with 95% confidence intervals (CI). A total of 3 studies met the inclusion criteria, providing data from 10,705,976 patients. A total of 28,100 patients were in the PCI + Cirrhosis group and 10,677,876 patients were in the PCI-only group. The mean age of patients with PCI + Cirrhosis and PCI alone was 63.45 and 64.35 years. The most common comorbidity was hypertension among the PCI + Cirrhosis group compared with PCI alone (68.15% vs. 73.6%). Cirrhosis patients post-PCI were had higher rates of in-hospital mortality (OR, 4.78 (95%CI: 3.39–6.75), *p* < 0.001), GI bleeding (OR, 1.91 (95%CI:1.83–1.99), *p* < 0.001, I^2^ = 0%), stroke (OR, 2.48 (95%CI:1.68–3.66), *p* < 0.001), AKI (OR, 3.66 (95%CI: 2.33–6.02), *p* < 0.001), and vascular complications (OR, 1.50 (95%CI: 1.13–1.98), *p* < 0.001) compared with the PCI group without cirrhosis. Patients with cirrhosis are at a high risk for mortality and adverse outcomes post-PCI procedure compared to the PCI-only group of patients.

## 1. Introduction

Cardiovascular disease (CVD) is the leading cause of mortality worldwide. Coronary artery disease (CAD), accounting for a major portion of it, contributes significantly to the disease burden, as assessed by disability-adjusted lifespan [1]. The emergence and growth of percutaneous coronary intervention (PCI) in the last decade have had a profound impact on the management of CAD [2]. With the evolution of PCI techniques over the years, PCI is now frequently offered to high-risk patients with comorbidities, including liver disease [3,4].

Recent data suggest an increasing prevalence of cirrhosis among US adults [5,6,7]. The prevalence of CAD is high in patients with cirrhosis [8,9], and CVD accounts for one of the major causes of mortality and morbidity in patients with end-stage liver disease [10]. Risk factors for atherosclerosis, such as obesity, diabetes, and metabolic syndrome, are common in cirrhosis patients, particularly in those with non-alcoholic steatohepatitis (NASH) [11]. Owing to the high surgical risk, and increased mortality and morbidity after coronary artery bypass grafting (CABG), PCI is preferred in patients with cirrhosis and CAD [12,13]. Underlying coagulation abnormalities, thrombocytopenia, and kidney injury, which are common in cirrhosis, confer increased risks of periprocedural bleeding, need for blood transfusions, and pseudoaneurysm formation [14]. The outcomes of PCI in patients with cirrhosis are scarcely studied. The objective of this meta-analysis was to study outcomes in patients with cirrhosis undergoing PCI. To the best of our knowledge, this is the first meta-analysis on the outcomes of PCI in patients with cirrhosis.

## 2. Materials and Methods

This systematic review was conducted and reported following the Cochrane and PRISMA (Preferred Reporting Items for Systematic Review and Meta-Analysis) 2020 guidelines, as described previously [15,16,17]. A pre-specified study protocol has been registered in the PROSPERO (CRD42022380609).

### 2.1. Outcomes of Interest

The primary outcome of this meta-analysis was in-hospital mortality. The secondary outcomes were stroke, acute kidney injury (AKI), vascular complications, and GI bleeding.

### 2.2. Search Strategy and Study Selection

We conducted a systematic search in PubMed, Embase, Scopus, and Cochrane Central for articles from their inception until 20 September 2022, using the following keywords: cirrhosis, liver cirrhosis, percutaneous coronary intervention, PCI, revascularization, and keywords for specific outcomes. MeSH terms were used where appropriate.

Eligible articles were assessed for methodological quality. Two authors (M.S. and A.T.) reviewed the abstracts and titles of the articles for eligibility. The senior author (H.K.) resolved any inclusion-related discrepancies. Studies that were included had all the following parameters:

- Patients with diagnosed cirrhosis;
- Studies with patients > 18 years;
- Two-arm studies comparing the PCI patients with cirrhosis and PCI patients without cirrhosis;
- Studies reporting at least one of the desired outcomes;
- Prospective and retrospective studies were eligible.

We excluded literature or systematic reviews, letters, single-arm studies, animal studies, and studies including patients < 18 years of age.

### 2.3. Data Extraction and Statistical Analysis

Data from the eligible studies, such as demographics, study design, comorbidity, follow-up, and short-term outcomes between cirrhosis and non-cirrhosis groups of patients, were extracted to a spreadsheet by two authors (V.J and A.I).

Baseline continuous variables were summarized as means (standard deviation), whereas dichotomous variables were described as frequencies or percentages. We performed a conventional meta-analysis for primary and secondary outcomes and adopted the DerSimonian and Laird random-effect model for the study variations [18]. Outcomes were reported as pooled odds ratio (OR), standard mean difference (SMD), and their corresponding 95% confidence interval (95% CI). Statistical significance was met if the 95% CI did not cross the numeric “1” and the two-tailed *p*-value was less than 0.05. We considered a two-tailed *p*-value of less than 0.05 to be statistically significant. In addition, we assessed the between-study heterogeneity using the Higgins I-square (I^2^) test, with I^2^ values < 75% considered mild to moderate and >75% considered high [19]. All statistical work, inclusive analysis, and graphical illustrations were conducted using STATA (version 17.0, StataCorp, College Station, TX, USA).

### 2.4. Quality Assessment

A.I independently assessed the quality of the included studies using the Newcastle–Ottawa Scale for cohort studies [20].

## 3. Results

The preliminary database search using the pre-specified keywords yielded 231 articles. Of these, 98 duplicate studies were excluded, and 110 studies were further excluded from the initial post-title and abstract screening based on the inclusion and exclusion criteria and comparison arm. A full-text review was conducted for the remaining 23 studies. Of these, 20 were excluded as they either had unmatching target populations, were not primary research articles or case reports, or lacked a comparison arm. Hence, three studies that met the eligibility criteria were included in our study [21,22,23]. The Preferred Reporting Items for Systematic Reviews and Meta-Analyses (PRISMA) flow diagram is depicted in Figure S1.

A total of 10,705,976 patients were included in the final analysis, of which 28,100 patients were in the PCI + Cirrhosis group and 10,677,876 patients were in the PCI-only group. The mean age of patients with PCI and cirrhosis and that of those in the PCI-only group were 63.45 and 64.35 years, respectively. The most common comorbidities were hypertension (68.15% vs. 73.6%) diabetes mellitus (47.45% vs. 34.9%), and hyperlipidemia (47.65% vs. 66.55%). The study characteristics, patient demographics, and comorbidities are presented in Table 1.

**Figure 1:**
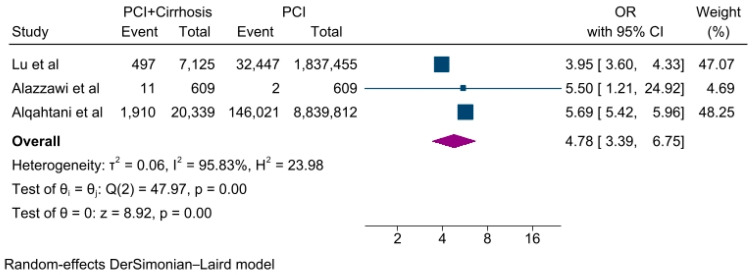
Forest plot of primary outcome: in-hospital mortality [21,22,23].

A meta-analysis of the primary outcomes showed that the odds of in-hospital mortality were higher in the PCI + Cirrhosis group compared with PCI without cirrhosis group (OR, 4.78 (95%CI: 3.39–6.75), *p* < 0.001, I^2^ = 95.83%) (Figure 1).

**Figure 2:**
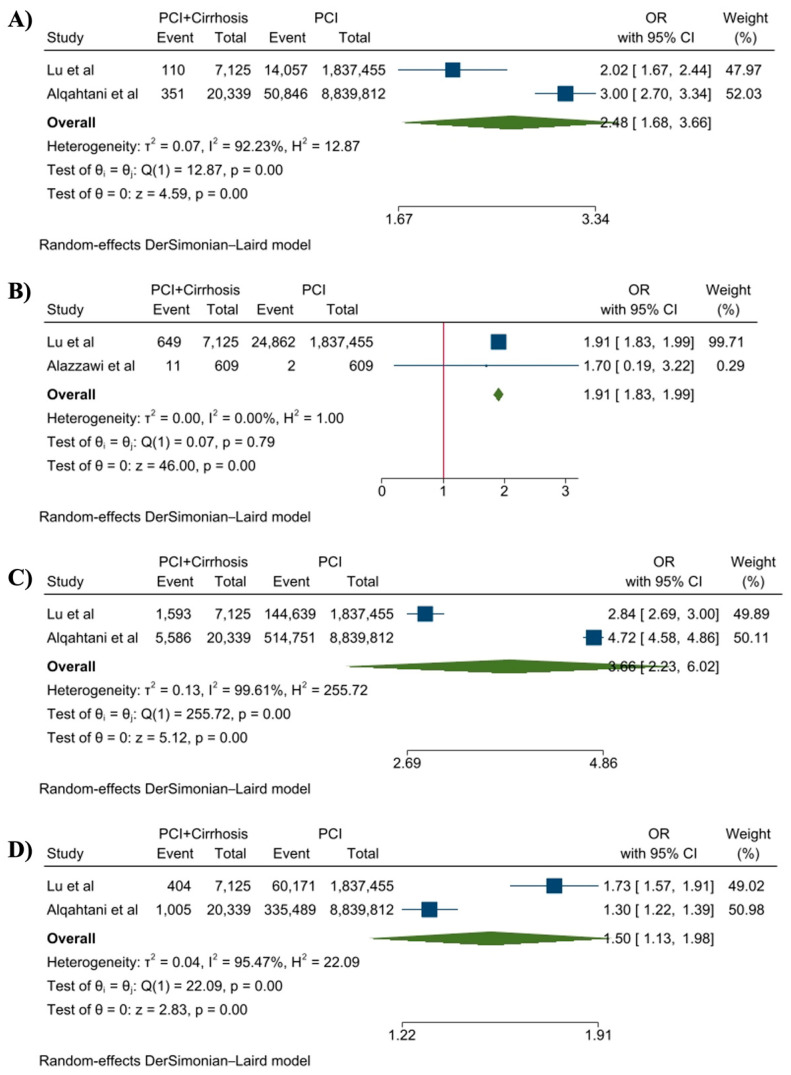
Forest plot of secondary outcomes: (**A**) stroke, (**B**) GI bleeding, (**C**) AKI, and (**D**) vascular complications [21,22,23].

The pooled analysis of secondary outcomes shows that the likelihood of stroke (OR, 2.48 (95%CI:1.68–3.66), *p* < 0.001, I^2^ = 92%), GI bleeding (OR, 1.91 (95%CI:1.83–1.99), *p* < 0.001, I^2^ = 0%), AKI (OR, 3.66 (95%CI: 2.33–6.02), *p* < 0.001), and vascular complications (OR, 1.50 (95%CI: 1.13–1.98), *p* < 0.001) were significantly higher among the cirrhosis group of patients compared to the non-cirrhosis group who underwent treatment for PCI (Figure 2A–D). The details of the quality assessment are presented in Table S1. All included studies had a low risk of bias.

## 4. Discussion

To the best of our knowledge, this is the first meta-analysis conducted to evaluate the outcome of cirrhotic patients in a group undergoing PCI. In our study, in-hospital mortality, gastrointestinal bleeding, vascular complications, stroke, and AKI were found to be higher in cirrhotic patients in comparison to non-cirrhotic patients after undergoing PCI.

Higher in-hospital mortality and vascular complications were reported in cirrhotic patients in comparison to non-cirrhotic patients who underwent PCI by Alqahtani et al., Lu et al., and Alazzawi et al., and the findings were concordant with our results [21,22,23]. Post-PCI gastrointestinal bleeding incidents were found to be higher in the PCI with cirrhosis group in comparison to the control group by Lu et al. and Alazzawi et al., corresponding with our study findings [21,22]. Similarly, AKI and post-PCI stroke events were reported to be higher in the cirrhotic group in comparison to the non-cirrhotic group by Alqahtani et al. and Lu et al., and the results were concordant with our findings [21,23]. Another study conducted by Kolte et al., using national inpatient sampling (NIS) from 2003–2011, reported a higher incidence rate of in-hospital mortality and gastrointestinal bleeding in the post-PCI cirrhotic group in comparison to non-cirrhotic patients, thus supporting the findings of our study [24].

The outcome of coronary interventions in patients with liver cirrhosis was studied by Marui et al., and they compared PCI with conventional on-pump coronary artery bypass graft surgery (CABG) [25]. There was a higher incidence rate of in-hospital mortality (6.9% vs. 0.4%), gastrointestinal bleeding (1.9% vs. 0.9%), and stroke (7.4% vs. 3.9%) in the CABG group in comparison to those who underwent PCI [25]. However, all-cause mortality (16.7% vs. 18.5%), myocardial infarction (3.7% vs. 4.3%), and revascularization (13.0% vs. 40.9%) were reported to be lower in those cirrhotic patients who underwent CABG in comparison to PCI [25].

The access site complications rate between the trans-radial approach and trans-femoral approach was studied by Feng et al., and the findings showed that the trans-radial approach was safer in terms of the complication rate in comparison to the trans-femoral approach in end-stage liver disease patients (ESLD) [26]. The study reported a lower rate of pseudoaneurysm (0% versus 3.7%, *p* = 0.0192) and hematoma (2.1% versus 3.7%, *p* = 0.3849) in the radial group in comparison to the femoral group with ESLD [26]. A hematocrit drop was found, which was significantly lower in the radial group in comparison to the femoral group (5.4% versus 7.8%, *p* = 0.0393), although no case of intracranial bleeding or retroperitoneal bleeds was reported in either group [26].

Singh et al. conducted a study in which overall mortality and complication rates, stratified by stent type in post-PCI cirrhotic patients, were studied using national inpatient samples (NIS) from 2005 and 2012 [27]. They revealed that bare-metal stents (BMS) were associated with a higher rate of mortality (4.72% vs. 2.64%, *p* < 0.01) and other complications in comparison to drug-eluting stents (DES), proving that the DES stent is the one of choice to be used in cirrhotic patients undergoing PCI [27].

The inferior clinical outcome in cirrhotic patients undergoing PCI is likely to be multifactorial. Anemia, thrombocytopenia, and coagulopathy could be possible reasons for increased bleeding and mortality in post-PCI cirrhotic patients [28]. The liver is responsible for producing clotting factors, and this ability is impaired in CLD, in both severe and non-severe cases. This is why major bleeding was found at a significantly higher rate in both severe and non-severe CLD cases [28]. However, Ostojic et al. reported that the bleeding risk in liver cirrhosis is not the same due to variable clinical presentation of cirrhotic patients and depends upon coagulation abnormalities, the extent of thrombocytopenia, and complications arising from portal hypertension, primarily esophageal varices [29]. Therefore, it is recommended that every effort should be made to maintain thrombocyte count above >50 × 109/L and prevent variceal bleeding [29]. Additionally, thrombotic events are also more common in cirrhotic patients due to various factors, i.e., low levels of plasminogen, protein C, protein S, antithrombin, and increased levels of factor VIII and von Willebrand factors (VWF) in cirrhotic patients [30]. These are all reasons for the significantly increased post-PCI stroke events in cirrhotic patients in comparison to non-cirrhotic patients.

### Strengths and Limitations

The main strength of our study is that it is the first meta-analysis conducted on a large sample size to evaluate the clinical outcome in cirrhotic patients undergoing PCI and found significant differences in comparison to non-cirrhotic patients. In our study, significantly higher odds of in-hospital mortality, gastrointestinal bleeding, stroke, vascular complications, and AKI were reported in cirrhotic patients in comparison to non-cirrhotic patients, concluding that cirrhotic patients are more prone to develop complications after PCI.

The main limitation of our study was that only three studies were included in the final reports. One of the included studies had a very low number of patients and its results had wide confidence intervals; therefore, it was of limited value to the analysis [22]. Moreover, many of the studies included were observational in nature; therefore, there could be confounders that affected the present results. Therefore, various further studies are needed to address the specified questions. Certain important clinical outcomes were not reported in the included studies. These included short-term and long-term patient mortality, and specific post-procedural outcomes such as risk of myocardial infarction post-PCI, coronary revascularization, stent thrombosis, cardiovascular death, and overall bleeding risk. Additionally, specific subgroup analyses, such as patients with portal hypertension compared with those without or different causes of cirrhosis, was not possible due to limited data from primary studies. Finally, the studies had short-term follow-up periods (90 days); hence, studies assessing long-term outcomes are required. We recommend that new prospective studies analyze these outcomes in patients with cirrhosis who are undergoing PCI.

## 5. Conclusions

Patients with cirrhosis are at high risk for mortality and adverse outcomes post-PCI procedure compared to the PCI-only group of patients. Future studies must aim to find more robust data among these patients to strengthen the knowledge and awareness among physicians regarding two broad spectra of subspecialties.

## Figures and Tables

**Table 1:** Baseline characteristic of included studies arranged in the form of (PCI + Cirrhosis/PCI).

Author	Sample, n (PCI-Cirrhosis/PCI)	Female, n	Age, Years	DM, n	HTN, n	HLD, n	AF, n	MI, n	Stroke, n
Lu et al., 2020 [21]	7125/1,837,455	2183/605,115	63.9/64.7	3658/668,513	5145/1,364,546	3684/1,314,543	1125/214,220	1050/256,047	102/16,636
Alazzawi et al., 2017 [22]	609/609	256/329	60.16/60	-	-	-	-	-	-
Alqahtani et al., 2020 [23]	20,366/8,839,812	5386/2,965,457	63/64	8882/2,962,452	13,064/6,139,439	6797/5,451,654		-	-

## Data Availability

The data presented in this study are available in the article and its supplementary materials.

## Supplementary Materials

The following supporting information can be downloaded at: https://www.mdpi.com/article/10.3390/jcdd10030092/s1, Figure S1: Preferred Reporting Items for Systematic review and Meta-analysis; flow of the search strategy for systematic review and meta-analysis; Table S1: Newcastle–Ottawa Scale for risk of bias assessment for cohort studies.
