# Supplementary material for: Percutaneous Coronary Intervention Outcomes in Patients with Liver Cirrhosis: A Systematic Review and Meta-Analysis
Source: J Cardiovasc Dev Dis. 2023 Feb 21;10(3):92. doi: 10.3390/jcdd10030092 (PMC10059068; doi:10.3390/jcdd10030092)
Supplement: Supplementary file 1 [file jcdd-10-00092-s001.zip › jcdd-2165927-supplementary.pdf]

Table S1. Newcastle-Ottawa Scale for risk of bias assessment for cohort studies.

Author	Selection n	Comparability	Outcome	Total
Lu et al [21]	**	**	***	7/9
Alazzawi et al [22]	***	**	***	8/9
Alqahtani et al [23]	**	**	***	7/9

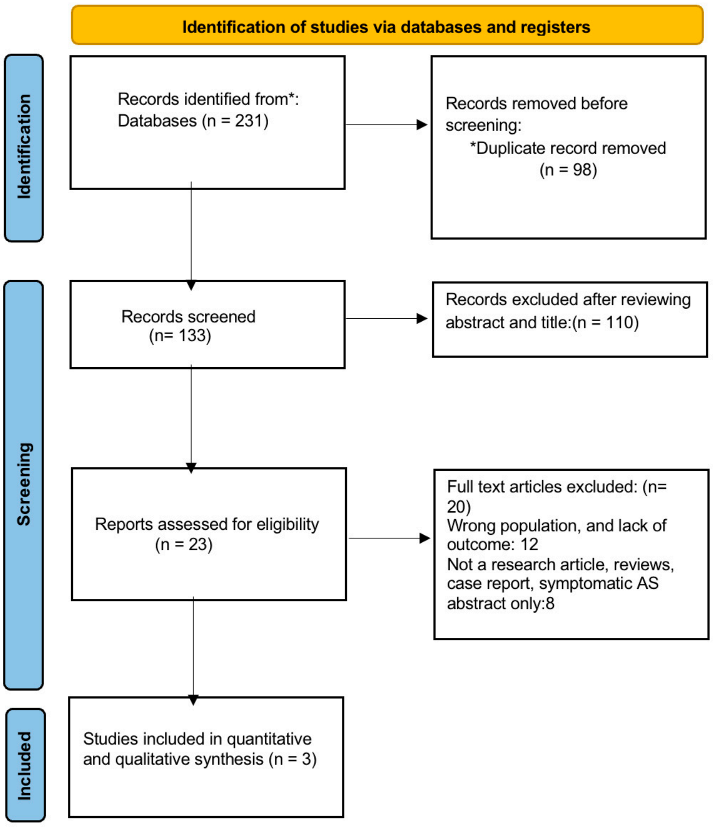

Figure S1. Preferred Reporting Items for Systematic review and Meta-analysis flow of the search strategy for systematic review and meta-analysis.

Outcome definitions:

Stroke: A stroke, sometimes called a brain attack, occurs when something blocks blood supply to part of the brain or when a blood vessel in the brain bursts. (CDC)

GI bleeding: Gastrointestinal (GI) bleeding is any type of bleeding that starts in the GI tract. GI bleeding is a symptom of a disease or condition, rather than a disease or condition itself.

AKI: KDIGO defines AKI as any of the following: Increase in serum creatinine by 0.3mg/dL or more within 48 hours or. Increase in serum creatinine to 1.5 times baseline or more within the last 7 days or. Urine output less than 0.5 mL/kg/h for 6 hours.

Vascular complications: Vascular disease includes any condition that affects your circulatory system, or system of blood vessels
